# FACTORS IN ABDOMINAL OBESITY, SARCOPENIA, AND SARCOPENIC OBESITY AMONG KOREAN ELDERLY: KNHANES 2022

**DOI:** 10.1093/geroni/igae098.3976

**Published:** 2024-12-31

**Authors:** Do Thi Thu Huyen, Xianqi Gao, Moonkyoung Park

**Affiliations:** Chungnam National University, Daejeon, Republic of Korea; Chungnam National University, Daejeon, Republic of Korea; Chungnam National University, Daejeon, Republic of Korea

## Abstract

**Background:**

Abdominal obesity, sarcopenia, and sarcopenic obesity are prevalent among the elderly, contributing to significant health risks. Understanding the nutritional, lifestyle, and biomarker profiles associated with these conditions is crucial for developing targeted interventions.

**Purpose:**

This study explores the associations between nutritional intake, lifestyle factors, and biomarkers with abdominal obesity, sarcopenia, and sarcopenic obesity among Korean elderly individuals, using data from the 2022 Korea National Health and Nutrition Examination Survey (KNHANES).

**Methods:**

The analysis included 845 participants (293 normal, 360 with abdominal obesity, 160 with sarcopenia, and 32 with sarcopenic obesity). Variables analyzed included hand grip strength, body composition, dietary habits (e.g., caloric intake, protein intake, supplement use), lifestyle factors (e.g., physical activity, sedentary behavior, smoking), psychological factors (e.g., depression, anxiety, stress), and biomarkers (e.g., hsCRP, cholesterol, triglycerides, blood pressure). Statistical analyses were conducted using SPSS 29.0, applying descriptive statistics and logistic regression within a complex sampling design.

**Results:**

The prevalence of abdominal obesity, sarcopenia, and sarcopenic obesity was 34.7%, 43.6%, 18.0%, and 3.8%, respectively. Sarcopenia was more prevalent among males, those aged 75 and older, and those living alone. Abdominal obesity was more common among those with hypertension or diabetes. Sarcopenic obesity was associated with living alone, high hsCRP, stress, and low physical activity.

**Conclusion:**

Comprehensive nutritional and lifestyle assessments are essential in managing abdominal obesity, sarcopenia, and sarcopenic obesity among the elderly. Public health strategies that improve diet quality and promote physical activity may mitigate these conditions.

